# Asymmetric (ADMA) and Symmetric (SDMA) Dimethylarginines in Chronic Kidney Disease: A Clinical Approach

**DOI:** 10.3390/ijms20153668

**Published:** 2019-07-26

**Authors:** Elena Oliva-Damaso, Nestor Oliva-Damaso, Francisco Rodriguez-Esparragon, Juan Payan, Eduardo Baamonde-Laborda, Fayna Gonzalez-Cabrera, Raquel Santana-Estupiñan, Jose Carlos Rodriguez-Perez

**Affiliations:** 1 Department of Nephrology, Hospital Universitario de Gran Canaria Doctor Negrín, 35010 Las Palmas de Gran Canaria, Spain; 2 Department of Medicine, Division of Nephrology, Hospital Costa del Sol, 29603 Marbella, Spain; 3 Department of Investigation, Hospital Universitario de Gran Canaria Doctor Negrín, 35010 Las Palmas de Gran Canaria, Spain; 4 Department of Medical and Surgical Sciences, Universidad de Las Palmas de Gran Canaria, 35001 Las Palmas de Gran Canaria, Spain

**Keywords:** ADMA, SDMA, methylarginines, asymmetric dimethylarginine, symmetric dimethylarginine, cardiovascular, chronic kidney disease, end-stage renal disease, uremia

## Abstract

Asymmetric dimethylarginine (ADMA) and its enantiomer, Symmetric dimethylarginine (SDMA), are naturally occurring amino acids that were first isolated and characterized in human urine in 1970. ADMA is the most potent endogenous inhibitor of nitric oxide synthase (NOS), with higher levels in patients with end-stage renal disease (ESRD). ADMA has shown to be a significant predictor of cardiovascular outcome and mortality among dialysis patients. On the other hand, although initially SDMA was thought to be an innocuous molecule, we now know that it is an outstanding marker of renal function both in human and in animal models, with ESRD patients on dialysis showing the highest SDMA levels. Today, we know that ADMA and SDMA are not only uremic toxins but also independent risk markers for mortality and cardiovascular disease (CVD). In this review, we summarize the role of both ADMA and SDMA in chronic kidney disease along with other cardiovascular risk factors.

## 1. Introduction

Asymmetric dimethylarginine (ADMA) and its enantiomer, symmetric dimethylarginine (SDMA), are naturally occurring amino acids that are generated intracellularly. They are post-translationally modified forms of arginine generated during normal protein turnover and were first isolated in human urine in 1970 by Kakimoto and Akazawa [1]. Since then, there have been numerous publications on these two molecules and their relationship with various diseases, especially with cardiovascular diseases. ADMA is the most potent endogenous inhibitor of nitric oxide synthase (NOS) and, therefore, inhibits the production of nitric oxide (NO), which is a key regulator of vascular tone. It has been shown to have a key role in endothelial dysfunction, as well as in the progression of atherosclerosis, and hence in cardiovascular disease. ADMA levels are higher in patients with end-stage renal disease (ESRD) compared to healthy individuals [2]. Among dialysis patients, ADMA may be a significant predictor of cardiovascular outcome and mortality [3]. On the other hand, although initially SDMA was thought to be an innocuous molecule, we now know that it is an outstanding marker of renal function, with patients with ESRD on dialysis showing the highest SDMA levels [4]. Both ADMA and SDMA are considered uremic toxins by the European Uremic Toxin Work Group (EUTox) [5]. Today, we know that ADMA and SDMA are independent risk markers for all-cause mortality and cardiovascular disease (CVD) [6].

In this review, we will summarize ADMA and SDMA metabolism, as well as their role in chronic kidney disease along with other cardiovascular risk factors and mortality. We will also review the role of ADMA in relation to other factors, such as endothelial dysfunction and inflammation, to better understand its role in cardiovascular disease and other factors such as hypertension and ageing. Finally, we will revise the relationship existing between ADMA and medication and the possibility of modifying ADMA levels with certain drugs, raising the possibility of ADMA being a modifiable and treatable risk factor, which could have clinical relevance in the future.

## 2. ADMA and SDMA Metabolism

**Figure 1:**
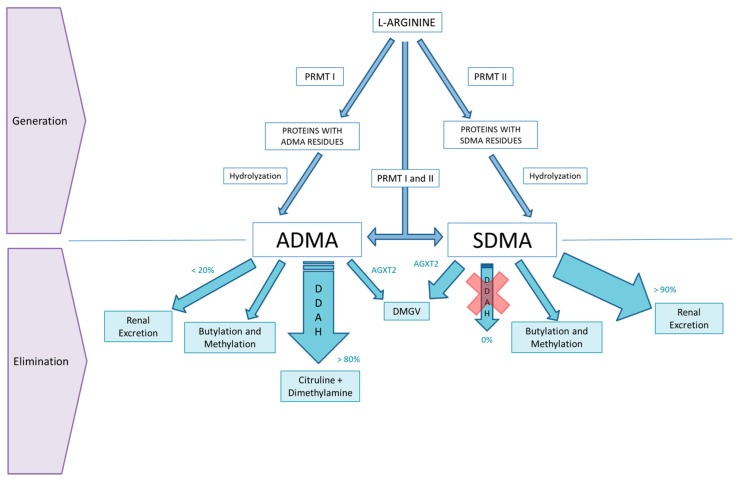
ADMA and SDMA metabolism. Both asymmetric dimethylarginine (ADMA) and symmetric dimethylarginine (SDMA) are generated basically by endogenous formation [2]. ADMA and SDMA originate from the monomethylation of protein-bound L-arginine by type I and II protein arginine methyltransferases (PRMTs), as well as by the liberation of ADMA and SDMA by protein degradation. However, ADMA is also generated by asymmetric dimethylation of protein-bound monomethylarginine by type I PRMTs, while SDMA also derives from asymmetric demethylation of protein-bound monomethylarginine by type II PRMTs [11]. There may also be an exogenous or dietary uptake source of ADMA and SDMA, but its exact contribution is unknown [6]. ADMA is excreted mainly by metabolism by dimethylarginine dimethylaminohydrolase (DDAH) 1 and 2 [12], and to a lesser degree by alanine glyoxylate aminotransferase 2 (AGTX2) [13], butylation, and methylation [14], while renal excretion represents a secondary and less important route of excretion [15]. SDMA’s major route of elimination is renal excretion, with metabolism being a minor route of elimination [15]. SDMA is not a substrate for DDAH so no SDMA is removed by DDAH. Alanine glyoxylate aminotransferase 2 (AGXT2) is the major metabolizing enzyme of SDMA [13] to DMGV (α-keto-δ-(N, N-dimethylguanidino) valeric acid), also contributing butylation and methylation [14].

When first described, Kakimoto and Akazawa already noticed that despite ADMA and SDMA being analogues of L-arginine, the overload of the latter was not shown to increase the elimination of ADMA and SDMA. They therefore deduced that ADMA and SDMA were not generated in a direct manner from the free amino acid [1]. Now we know that these molecules are released from proteins that are largely found on the nucleus and that have been post-translationally methylated and later hydrolyzed (Figure 1).

Initially, it was considered that ADMA’s main route of elimination was via renal excretion, based on the fact that ADMA levels were increased in patients with end-stage renal disease [2]. However, posterior investigations led to the identification of dimethylarginine dimethylaminohydrolase (DDAH), an enzyme that favors hydrolysis of ADMA to dimethylamine and L-citrulline [7]. DDAH is the key enzyme which regulates ADMA levels in tissues and intracellularly, and it is expressed predominantly in the kidney, brain, pancreas, and liver. DDAH I is present predominantly in tissues that express neuronal nitric oxide synthase (NOS), while DDAH II is found in tissues that express endothelial NOS [7]. Finally, ADMA can also decrease by transamination, by an enzyme called alanine glyoxylate aminotransferase 2 (AGXT2), to DMGV (α-keto-δ-(N, N-dimethylguanidino) valeric acid), which is a mitochondrial aminotransferase that is primarily expressed in the kidneys [8] (Figure 1). Therefore, the kidneys play a central role in the elimination of ADMA by excretion in the urine and also via metabolization by DDAH and AGXT2. This could explain why patients with higher levels of proteinuria have higher levels of ADMA, independent of glomerular filtration rate (GFR) [9], or why ADMA arises early in patients with autosomal dominant polycystic kidney disease (ADPKD) or IgA nephropathy [10].

SDMA, however, is removed almost exclusively via renal excretion [1], although AGXT2 can also metabolize SDMA, in contrast to DDAH which does not contribute to SDMA metabolization (Figure 1). This might be an explanation for the finding that SDMA is a better marker than CKD-EPI (Chronic Kidney Disease Epidemiology Collaboration) and MDRD(Modification of Diet in Renal Disease)-derived estimated glomerular filtration rate (GFR) equations for the assessment of renal function in certain patient groups [16] and also seems to be a precocious marker of GFR change after living-related kidney donation [17]. However, the elimination of SDMA and ADMA is not only dependent on the kidneys. The liver is also capable of clearing an important part of these molecules [18,19,20].

## 3. SDMA, Mortality and Cardiovascular Disease

Although SDMA was not considered biologically active until recently, in a meta-analysis, Schlesinger et al. [6] showed that SDMA, as well as ADMA, is an independent risk marker for all-cause mortality and cardiovascular disease. Patients with higher SDMA levels are at higher risk of dying from any cause, with this association being stronger in the general population and weaker in patients with established cardiovascular disease. In patients with renal failure, however, the association was not statistically significant, probably due to the fact that SDMA is a very sensitive marker to changes in renal function [6].

Regarding CVD, SDMA was associated with a higher CVD risk increase, which was, again, stronger in the general population in comparison to other groups [6].

## 4. SDMA and Chronic Kidney Disease

As SDMA is excreted mainly by the kidneys, various parameters of renal function show a close relationship with SDMA. SDMA is an excellent marker of kidney function, more precocious than CKD-EPI and MDRD-derived eGFR for the evaluation of renal function in certain population groups [16,17].

**Figure 2:**
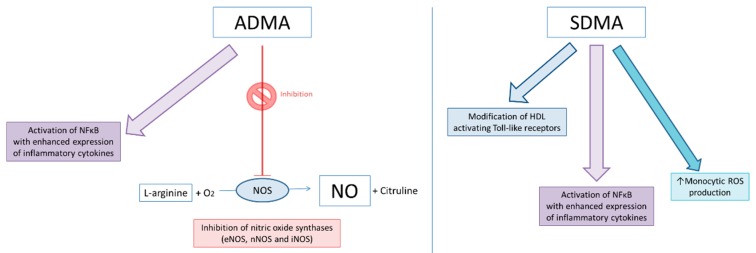
Biological effects of ADMA and SDMA. ADMA’s most relevant known biological effect is the inhibition of nitric oxide synthases [endothelial nitric oxide synthase (eNOS), neuronal nitric oxide synthase (nNOS) and inducible nitric oxide synthase (iNOS)] [2]. However, it has also been shown to induce activation of NFκB, with enhanced expression of inflammatory cytokines [31] and a weak inhibition of L-arginine transport [32]. SDMA does not inhibit nitric oxide synthases [2], but may have a possible weak indirect inhibition [24]. Like ADMA, SDMA produces activation of NFκB with enhanced expression of inflammatory cytokines [31] and a weak inhibition of L-arginine transport [32]. However, SDMA differs from ADMA by increasing monocytic reactive oxygen species (ROS) production by activation of calcium channels [33] and modification of high-density lipoprotein (HDL), activating Toll-like receptors [22].

SDMA in chronic kidney disease (CKD) patients was more significantly correlated to interleukin (IL)-6 and tumor necrosis factor-alpha (TNF-α) levels compared to ADMA [21]. SDMA also modified high-density lipoprotein (HDL), inducing endothelial damage [22] (Figure 2). In the last decade, there have been several studies which show an association between increased SDMA levels and cardiovascular risk factors and worse outcome [13,21,23,24,25,26,27,28,29,30].

Although SDMA is related to older age, it is also increased in hypertensive adolescents [34] and children with CKD [35], and also correlated in both cases with renal function. High SDMA levels also seem to play an important role in the progression to end-stage renal disease in CKD patients [28]. Recently, the potential pathophysiological role of SDMA in CKD progression and atherosclerotic cardiovascular disease among non-dialysis CKD patients has been analyzed, showing that SDMA could predict CKD progression and atherosclerotic cardiovascular events better than other methylarginines could [36].

However, how dialysis affects SDMA levels is not well known. Anderstam et al. [37] described that even though both molecules have identical molecular weight, a single dialysis session could reduce SDMA plasma levels more efficiently than ADMA levels (40% vs. 23%) [18,37].

## 5. ADMA, Mortality and Cardiovascular Disease

Multiple studies have linked circulating ADMA concentrations to cardiovascular disease (CVD) risk and mortality, following the studies by Zoccali et al. [3] and Valkonen et al. [38] in 2001. Cohort studies in patients with CKD and in general population showed a strong and independent link between ADMA, all-cause mortality and cardiovascular events [3,25].

There seems to be a strong association between levels of ADMA and prevalent CVD, and a weaker association with all-cause and CVD mortality in patients with nondiabetic CKD (stages 3 to 4) [39]. However, in the same study the researchers did not find any association with kidney failure or the composite outcome [39].

In their meta-analysis, Schlesinger et al. [6] confirmed a strong association between ADMA and all-cause mortality, with an approximately 50% increased risk of all-cause mortality in patients with higher ADMA levels, especially in critically ill patients, with a non-linear relation between ADMA and all-cause mortality [6].

When analyzing CVD, they observed a risk increase of 33% in patients with higher ADMA levels, with a positive association between ADMA and CVD [6].

## 6. ADMA and Chronic Kidney Disease

It is known that ADMA is significantly increased in ESRD [40], as well as in hypertension, adverse cardiovascular events [3,37,41,42], progression of kidney failure [43], renal fibrosis [44], and mortality [3].

In 1992, Vallance et al. showed that patients with ESRD on hemodialysis had higher ADMA levels than controls [2]. Among the ESRD patients on dialysis, ADMA seems to predict cardiovascular outcome and mortality [3].

Investigations have found that NO synthesis is inhibited in patients with end-stage renal disease (ESRD) and that ADMA is capable of inhibiting NO production in vivo and in vitro [2], which may result in vasoconstriction, hypertension and immune dysfunction, among other outcomes. We also know that increased ADMA levels are also associated to other cardiovascular risk factors such as increased plasma levels of low-density lipoprotein cholesterol, triglycerides, glucose, homocysteine, and mediators of inflammation [45].

There are studies, however, which refer to the association between treatment with erythropoietin (EPO) and ADMA levels. Scalera et al. found that in vitro EPO post-translationally impairs DDAH activity via increased oxidative stress and that endothelial cells responded to EPO by increasing the production of ADMA in a dose-dependent manner [46]. Although EPO has important beneficial effects in the correction of anemia, it has also been observed that it may have side effects, such as an increase in blood pressure (BP) and alteration in the production of nitric oxide in endothelial cells, thus increasing oxidative stress [47]. Again, Scalera et al. observed in patients with CKD, who received treatment with EPO for the first time, that there was an increase in plasma ADMA levels [46]. More recently it has been observed that erythrocyte ADMA accumulation may suppress erythropoietin receptor expression, contributing to impaired response to EPO in pre-dialysis patients [48].

## 7. ADMA and Dialysis

Because ADMA, like urea, has a low molecular weight, dialysis seems to be the best option for the elimination of ADMA in patients with CKD [18]. However, studies that have evaluated the impact of hemodialysis (HD) on plasma ADMA concentrations show that this view is too simplistic, since the reduction of ADMA does not necessarily translate to a subsequent clinical significance [18]. Some studies have shown a significant decrease in plasma ADMA concentrations with HD, which ranged from 23% [37] to 65% [49]. However, other studies did not find a significant decrease in ADMA levels post-dialysis [40,50,51]. The data suggest that dialysance, and therefore the elimination of ADMA by dialysis, is hampered by the fact that ADMA is bound to plasma proteins [51]. Only about 12% of the amount of ADMA produced per day was detected in the dialysate after a standard HD [51]. Although there are small studies suggesting that high-flux membranes and hemodiafiltration [52] may be more efficient to eliminate ADMA, this has not been confirmed [53,54].

## 8. ADMA and Hypertension

Regarding the relationship between ADMA and hypertension, there is increasing evidence that NO plays a relevant role in the regulation of vascular tone and BP [55,56]. Two possible mechanisms might explain how ADMA participates in the pathogenesis of hypertension. On the one hand, ADMA, through the inhibition of endothelial NOS activity, can exert a vascular vasoconstrictor effect [57,58]. On the other hand, ADMA can inhibit the renal excretion of sodium by reducing renal NO synthesis [59,60,61]. Kielstein et al. showed that exogenous ADMA increases systemic vascular resistance, as well as mean arterial pressure, and reduces cardiac output in men [42]. In this study, the administration of ADMA dose-dependently impaired renal blood flow and sodium reabsorption.

In rats undergoing 5/6 nephrectomy, ADMA levels are clearly related to BP levels [62]. In human Japanese subjects without coronary artery disease or peripheral arterial disease, it has been observed that plasma levels of ADMA are associated with higher mean BP levels [63]. This relationship seems to be more evident in patients with essential hypertension [38,64,65], especially in those individuals who are salt sensitive [66]. ADMA is probably the most important regulator of NO in the kidney [67]. A high intake of salt increases the urinary excretion of ADMA [68] and also increases the expression of all isoforms of NOS in the renal medulla [69]. On the other hand, BP itself can raise plasma ADMA levels through the positive regulation of protein arginine methyltransferases (PRMTs) by an increase in shear stress through the NFκB pathway [70]. Plasma levels of ADMA rise in response to an expansion of extracellular volume. Thus, in hypertensive patients, in the presence of salt overload, an increase in ADMA levels and a decrease in NO levels can be observed [66]. Likewise, angiotensin II and the generation of reactive oxygen species (ROS) may also be involved in the elevation of ADMA levels in hypertension [71]. This correlation between hypertension and ADMA levels is quite clear in subjects with essential hypertension or with some degree of CKD, where the regulation of BP through the kidneys is still preserved. However, in hemodialysis patients without residual renal function, we do not have reliable data about the relationship between ADMA and hypertension.

## 9. ADMA and Ageing

Several studies have found that plasma ADMA levels increase with age [63,72,73]. Some authors point out the possibility that ADMA increases with age due in part to the decrease in GFR associated with ageing [67,72]. Interestingly, an in vitro study demonstrated that ADMA increases the rate of endothelial senescence and shortens the telomere length by means of a significant reduction in telomerase activity in endothelial cells treated with ADMA, in comparison with the control group [74]. The authors concluded, therefore, that ADMA accelerates ageing and may increase oxidative stress in endothelial cells.

## 10. ADMA, Endothelial Dysfunction and Inflammation

Endothelial dysfunction measured by ADMA and inflammation has been clearly related to atherosclerosis, cardiovascular events and death in patients with CKD [75]. Inflammation is capable of amplifying the effect of ADMA on the severity of atherosclerosis in patients with CKD [75]. However, if ADMA and inflammation interact, it is still unknown whether this interaction increases the risk of cardiovascular events and death [75]. In a study by Tripepi et al. [75], in a cohort of 225 patients on hemodialysis, it was observed that there was an interaction between biomarkers of inflammation and ADMA, so that mortality was higher in the group that had both elevated ADMA and C-reactive protein (CRP), in comparison with groups that only had one of the markers elevated. The authors concluded, therefore, that inflammation amplifies the risk of death and cardiovascular events in patients with high ADMA levels in subjects on hemodialysis [75]. Other studies show that endothelial dysfunction is associated with chronic inflammation [76,77]. In patients with type 2 diabetes mellitus, an interaction between CRP and ADMA was observed in a prospective cohort study, which was also related to cardiovascular events [78]. However, the relationship between ADMA and CRP depends on the context, since in acute sepsis there is a decrease in ADMA levels [79].

Inflammation in patients with CKD is a multifactorial problem [79], since both factors related to dialysis and factors independent of it can favor inflammation by stimulating the synthesis or release of proinflammatory cytokines such as PCR, IL-1, IL-6, TNF-α, and IFN-γ [77].

## 11. ADMA and Drugs: A Modifiable Risk Factor?

It seems that there is a pathophysiological relation between the use of proton pump inhibitors (PPIs) and an increase in cardiovascular events, as it seems that these drugs have the ability to bind and inhibit DDAH, which results in higher ADMA levels and a decrease in NO bioavailability [80]. This could be a reasonable mechanism to explain the association of PPIs with an increase in major adverse cardiovascular events in certain patient groups. On the other hand, a cross-sectional analysis in patients with CKD on hemodialysis hypothesized a link between paricalcitol and ADMA levels [81]. Paricalcitol provides a survival benefit in patients with CKD [82,83], although the biological mechanism of this beneficial effect is not yet fully clarified. The PENNY trial (Paricalcitol and ENdothelial fuNction in chronic kidneY disease trial) [84] showed that paricalcitol improves endothelium-dependent vasodilatation in patients with stage 3–4 CKD. However, in a publication by the same group, the group found that paricalcitol did not modify plasma ADMA and SDMA in patients with stage 3–4 CKD [85].

Even more interesting is the fact that ADMA could be a potentially modifiable risk factor. Studies have shown that certain drugs (such as rosiglitazone, pioglitazone, amlodipine, valsartan, and targeted gene therapies) decrease ADMA levels in animal models and patients with kidney disease [86,87,88].

Considering the mechanism by which nitric oxide is formed, it has been considered to reverse the competitive inhibition of ADMA by the administration of L-arginine. However, in patients with kidney disease, studies are scarce, and the results have not been conclusive [89]. Although one study showed a positive effect on endothelial function, another failed to demonstrate this effect [89,90]. In a clinical trial in which L-arginine was added to the standard post-myocardial infarction treatment, not only was no benefit shown, but there was a trend towards higher mortality [91]. A similar result was observed in a study in patients with peripheral arterial disease, where L-arginine showed a detriment to vascular function in these patients, so this strategy has been set aside [92].

In a human study, perindopril, an angiotensin-converting enzyme (ACE) inhibitor, reduced plasma ADMA levels in 11 non-insulin-dependent diabetes mellitus patients [93]. Both enalapril and eprosartan reduced ADMA levels in 20 patients with essential arterial hypertension [94]. This result was confirmed with zofenopril and enalapril in a larger study with 96 patients diagnosed with essential hypertension [95]. However, in a randomized controlled study by Fliser et al., after 4 weeks of treatment with Olmesartan at a dose of 40 mg/day in 35 patients with non-insulin-dependent diabetes mellitus, no effect on plasma ADMA levels could be demonstrated [96]. Whether ACE inhibitors and/or Angiotensin II Receptor Blockers (ARBs) have a direct effect on ADMA metabolism, or whether the change in ADMA levels is a result of changes in blood pressure, is not yet clear.

In contrast to the contradictory data on the effect of ACE inhibitors and ARBs, oral antidiabetics have been shown to be more effective in reducing ADMA levels. ADMA levels were reduced after improvement in glycemic control with metformin in diabetic patients [97]. Rosiglitazone also reduced ADMA levels and improved insulin resistance in seven insulin-resistant patients with hypertension [88]. This effect is probably a consequence of the upward regulation of DDAH. In fact, in rats, rosiglitazone (PPARγ ligand) increases the production of nitric oxide, in part by the positive regulation of tissue DDAH II expression and by the decrease in systemic levels of ADMA [98]. This is probably due to a specific action of the PPARγ ligand on DDAH, rather than the effect of better glycemic control, since in studies in humans with acarbose, a better glycemic control was achieved but no changes were observed in the levels of ADMA [99].

On the other hand, estrogen therapy (alone or in combination with progestogens) reduced ADMA levels in postmenopausal women [100,101]. This was probably due to an increase in DDAH activity induced by estrogens [102]. Treatment aimed at reducing oxidative stress should also reduce ADMA levels [103]. Two preliminary studies showed a small beneficial effect of folic acid and vitamin E on ADMA levels [104,105]. More recently, ascorbic acid was shown to reduce serum ADMA in CKD [106].

Regarding the relationship between statins and ADMA, there are several studies about the effect of statins on ADMA levels. Neither atorvastatin, pravastatin nor simvastatin [107,108] reduce plasma ADMA levels. However, in a study carried out by Lu et al., rosuvastatin did achieve a reduction in ADMA [109]. It is believed that statins may possibly cause an increase in endothelial NO production in vascular cells, an increase in endothelial NO synthase and an increase in antioxidant systems, as possible underlying mechanisms [110,111,112]. Recently, in a large cohort of CKD patients, fenofibrate and glucocorticoids were independently associated with higher and lower plasma homoarginine concentrations, respectively [113].

The role of a specific drug, the farnesoid X receptor agonist GW 4064, is being investigated, given its ability to increase the expression of DDAH and decrease ADMA levels [114]. The farnesoid X receptor agonist increases biliary excretion of ADMA during hepatic ischemia/reperfusion injury [115].

So far, pharmacological targeting of ADMA or SDMA has not been demonstrated to be successful or clinically relevant. Theoretically, ADMA and SDMA levels could be reduced by decreasing endogenous generation or dietary uptake, or by increasing its elimination [6]. Currently, there is no clear evidence that ADMA levels can be reduced with medication, just as it is not clear whether or not the reduction of ADMA levels has any clinical relevance.

## 12. Conclusions

Since 1970, when ADMA and SDMA were first isolated from human urine, there has been important evidence demonstrating that these molecules play an important role in mortality and cardiovascular disease, as well as in the progression of CKD. ADMA and SDMA are markedly increased in patients with CKD. Although studies have focused on ADMA, the role of SDMA in CKD and as a cardiovascular risk factor is gaining evidence. ADMA is not just another uremic toxin but also a potent mediator of endothelial dysfunction and atherosclerosis, with physiological and pathological relevance that may be useful as a marker of early diagnosis in patients with a risk of progression of kidney disease, cardiovascular events, and mortality. Both ADMA and SDMA have been shown to be independent risk markers for CVD and all-cause mortality [6]. Currently, there is no clear evidence that ADMA and SDMA levels can be reduced with drugs, just as it is not clear whether or not the reduction of ADMA and SDMA levels has any clinical relevance or improves patient-centered outcomes. A deeper understanding of the effects of these methylarginines, the effect of lowering them, and the development of specific pharmacological therapy for lowering ADMA and SDMA levels, is still a future objective that requires further investigation [116].

## 13. Take-Home Messages

1. ADMA and SDMA are not only uremic toxins but also independent risk markers for all-cause mortality and cardiovascular disease.
2. Both ADMA and SDMA are increased in patients with end-stage renal disease, but SDMA is a much more sensitive marker to changes in renal function than ADMA, as it is removed almost exclusively by the kidneys.
3. SDMA is good marker of renal function, which has been shown to be better than CKD-EPI and MDRD-derived eGFR for the assessment of kidney function in certain population groups.
4. ADMA is also associated with ageing, hypertension, progression of kidney failure, and renal fibrosis.
5. ADMA could be a potentially modifiable risk factor as certain drugs could decrease ADMA levels. However, it is not yet clear whether this reduction will be of clinical relevance.
